# Occurrence, Antibiotic Susceptibility, Biofilm Formation and Molecular Characterization of *Staphylococcus aureus* Isolated from Raw Shrimp in China

**DOI:** 10.3390/foods12142651

**Published:** 2023-07-10

**Authors:** Jingsha Dai, Jiahui Huang, Shi Wu, Feng Zhang, Yuanyu Li, Dongli Rong, Miao Zhao, Qinghua Ye, Qihui Gu, Youxiong Zhang, Xianhu Wei, Jumei Zhang, Qingping Wu

**Affiliations:** 1 College of Food Science, South China Agricultural University, Guangzhou 510642, China; daijs@gdim.cn; 2 Guangdong Provincial Key Laboratory of Microbial Safety and Health, State Key Laboratory of Applied Microbiology Southern China, Key Laboratory of Agricultural Microbiomics and Precision Application, Ministry of Agriculture and Rural Affairs, Institute of Microbiology, Guangdong Academy of Sciences, Guangzhou 510070, China; jiahui0418@163.com (J.H.); wushiloveyou@126.com (S.W.); zfloveissun@163.com (F.Z.); iclyyu@163.com (Y.L.); rongdl69@126.com (D.R.); zm93032@163.com (M.Z.); yeqinghua2002@163.com (Q.Y.); guqh888@163.com (Q.G.); zyx0713141061@126.com (Y.Z.); wxhu7508@163.com (X.W.); zhangjm926@126.com (J.Z.)

**Keywords:** *Staphylococcus aureus*, shrimp, antibiotic resistance, biofilm formation, whole-genome sequencing

## Abstract

The aim of this study was to determine the prevalence and characterization of *Staphylococcus aureus* isolated from 145 shrimp samples from 39 cities in China. The results show that 41 samples (28%) from 24 cities were positive, and most of the positive samples (39/41, 95.1%) were less than 110 MPN/g. Antimicrobial susceptibility testing showed that only seven isolates were susceptible to all 24 antibiotics, whereas 65.1% were multidrug-resistant. Antibiotic resistance genes that confer resistance to β-lactams, aminoglycosides, tetracycline, macrolides, lincosamides and streptogramin B (MLSB), trimethoprim, fosfomycin and streptothricin antibiotics were detected. All *S. aureus* isolates had the ability to produce biofilm and harbored most of the biofilm-related genes. Genes encoding one or more of the important virulence factors staphylococcal enterotoxins (*sea, seb* and *sec*), toxic shock syndrome toxin 1 (*tsst-1*) and Panton–Valentine leukocidin (PVL) were detected in 47.6% (30/63) of the *S. aureus* isolates. Molecular typing showed that ST15-t085 (27.0%, 17/63), ST1-t127 (14.3%, 9/63) and ST188-t189 (11.1%, 7/63) were the dominant genetic types. The finding of this study provides the first comprehensive surveillance on the incidence of *S. aureus* in raw shrimp in China. Some retained genotypes found in this food have been linked to human infections around the world.

## 1. Introduction

With the development of the economy and improvement in people’s living standards in our country, cold chain transportation is continuously improving, and consumers all over our country can eat aquatic products rich in protein. Shrimp is one of the most popular aquatic products, and the estimated level of production from both wild harvest and farm culture is approximately 6624 million metric tons, totaling a value of more than USD 23 billion [1]. According to the China Fishery Statistics Yearbook, the shrimp production in China reached 6,307,300 tons in 2020, and the domestic production of shrimp essentially matches domestic consumption, with significant economic benefits. Shrimp is enjoyed for the uniqueness of its flavor and texture, but one of the major problems faced by the shrimp industry, besides insufficient production and disease outbreaks, is shrimp product safety [1]. In recent years, the prevalence and risk identification of some foodborne pathogens, such as *Vibrio parahaemolyticu, Listeria* or *Salmonella,* in shrimp have been reported frequently [1,2]. These pathogens and toxins found in shrimp can cause food poisoning and pose a risk to public health. Therefore, shrimp may play an important role in endangering public health.

*Staphylococcus aureus* is also one of the most important foodborne pathogens in the world. It is a type of bacteria commonly found on the skin and in the noses and throats of humans that can produce super-antigen exotoxin with different characteristics. It exists widely in nature, and can be found in water and the air [3]. Food contaminated by *S. aureus* can cause acute gastroenteritis symptoms such as diarrhea, vomiting and fever, or even necrotizing pneumonia, bacteremia, sepsis, toxic shock syndrome, etc. [4,5]. It has low nutritional requirements, is aerobic or facultatively anaerobic and has a high salt tolerance. It is well suited to survival in various water environments, and poses a great threat to the quality and safety of aquatic products. Research on *S. aureus* in shrimp has received little attention and deserves more focus.

Generally, *S. aureus* produces a variety of toxins and invasive enzymes, such as staphylococcus enterotoxins (SEs), Panton–Valentine leukocidin (PVL), toxic shock syndrome toxin-1 (TSST-1), hemolysis, plasma coagulase and deoxy ribonuclease [6,7]. Of these, SEs can cause food poisoning and endanger human health after consumption [8]. Some studies have shown that 0.02 ng/g SEs can cause food poisoning in susceptible people [9]. In addition, *S. aureus* biofilm has strong adhesion, environmental adaptability and self-protection, which means it is recognized as an important virulence factor for bacteria of the genus *Staphylococcus*. The formation of *S. aureus* biofilm is related to polysaccharide intercellular adhesin (PIA), and its synthesis is mainly regulated by *ica* genes, including *icaA*, *icaD*, *icaB*, *icaC* and regulatory genes *icaR* [10]. Agglutination factors A (*ClfA*) and B (*ClfB*), fibrinin-binding proteins (FnBPs), serine–aspartic acid repeating protein (Sdr) and collagen adhesion (*can*) have also been associated with biofilm formation [11,12,13,14]. In recent years, the widespread use of antibiotics has increased the emergence of multidrug-resistant strains, which have become a major public health concern. *S. aureus* is one of these major foodborne pathogens, having the formidable ability to adapt to varying environmental conditions and an extraordinary capacity to rapidly become resistant to virtually all antibiotics. Several recent studies have reported that food animals, meat, dairy and fishery products are contaminated by multi-resistant *S. aureus* strains and many food poisoning outbreaks are caused by multidrug-resistant (MDR) *S. aureus* [15,16,17,18,19]. Furthermore, the ability of some strains to synthesize biofilm could increase the pathogenicity of isolates, since established biofilms can tolerate antimicrobial agents, thus making the bacterium extremely difficult to eradicate [7,20,21].

In this study, *S. aureus* was selected as the object to study its contamination on shrimp samples from 39 cities in China and carry out risk identification research. The prevalence and levels of *S. aureus* in these samples, as well as antimicrobial susceptibility profiles and biofilm formation were investigated, and we analyzed the genetic background through whole-genome sequencing involving biofilm-related genes, *spa* typing and multi-locus sequence typing to determine the molecular characterization among the isolates.

## 2. Materials and Methods

### 2.1. Sampling Sites

A total of 145 raw shrimp samples were collected from supermarkets, fairs and farmers’ markets throughout the last decade (Supplementary Figure S1). The samples were obtained from 39 cities in 29 provinces and 2 directly controlled municipalities in China. These sample sites covered most of the provincial capitals of China, which are shown in Supplementary Figure S2. The samples were stored in a cold box at around 4 °C and sealed with sterile plastic wrap. They were then transported to an accredited laboratory within 24 h for microbiological analysis.

### 2.2. Isolation and Identification of S. aureus

The shrimp samples were submitted to qualitative and quantitative analysis for *S. aureus*. Qualitative analysis was performed according to the GB 4789.30-2010 of food microbiological examination of *S. aureus* (National Food Safety Standards of China) with slight modification, and quantitative analysis was performed with the most probable number (MPN) method. Briefly, approximately 25 g of food sample was added to 225 mL of tryptic soy broth culture with 10% sodium chloride (Huankai, Guangzhou, China). Subsequently, 1 mL, 0.1 mL and 0.01 mL aliquots were transferred to tubes containing 9 mL, 10 mL and 10 mL in triplicate with trypticase soy broth culture (Huankai) supplemented with 10% NaCl. Each tube was incubated at 37 °C for 48 h, respectively.

A portion (10 µL) of enrichment broth culture was streaked onto chromogenic *S. aureus* agar plates (Huankai), which were incubated at 37 °C for 24 h. Each positive sample selected 1–4 colonies with pink color and was purified on nutrient agar medium at 37 °C for 24 h. The purified colonies were analyzed via coagulase activity test by freeze-dried Rabbit Plasma (Huankai), and the API STAPH test strips (bio Merieux, Marcy-1′Etoile, France). The MPN value was determined based on the number of positive tube(s) in each of the three sets using the MPN table.

### 2.3. Antimicrobial Susceptibility Testing for Shrimp-Related S. aureus

To determine the antimicrobial susceptibility, all shrimp-related *S. aureus* isolates were tested with the Kirby–Bauer disk diffusion method. All isolates were assessed for antimicrobial susceptibility to 24 antibiotics (Oxoid, Basingstoke, Hampshire, UK) in 14 different groups: β-Lactams (amoxycillin/clavulanic acid, ampicillin, cefepime, cefoxitin, penicillin G, and ceftazidime), Aminoglycosides (amikacin, gentamicin, kanamycin, and streptomycin), Phenicols (chloramphenicol), Lincosamides (clindamycin), Macrolides (erythromycin, telithromycin), Fluoroquinolones (ciprofloxacin, norfloxacin), Tetracyclines (tetracycline), Oxazolidinones (linezolid), Sulfonamides (trimethoprim/sulphamethoxazole 1:19), Ansamycins (rifampicin), Quinolones (quinupristin/dalfopristin), Glycopeptides (teicoplanin), Nitrofurantoins (nitrofurantoin) and Fusidic acid. *S. aureus* ATCC25923 and *Escherichia coli* ATCC25922 were used as quality control organisms and the diameter interpretations were based on the protocol of the guidelines of the Clinical and Laboratory Standards Institute (CLSI, 2018). CLSI zone diameter breakpoints were used to interpret the antimicrobial susceptibilities of the analyzed strains.

### 2.4. Biofilm Formation Assay

The ability of *S. aureus* isolates to produce biofilm in vitro was assessed by a microtiter plate assay (MPA) as described by Vasudevan et al. (2003) [22] with slight modifications. The isolates were individually cultivated in 37 °C overnight on BHI (Brain Heart Infusion Broth). The overnight culture was diluted 1:100 in fresh BHI, and an aliquot of 200 µL of each prepared suspension were transferred into three wells of 96-well tissue-culture-treated polystyrene microplates (CELLSTAR^®^ Cell Culture Microplates, Greiner Bio-one, Frickenhausen, Germany). After cultivation at 37 °C for 48 h, the wells were washed three times with 200 mL of sterile phosphate-buffered saline (PBS, pH 7.4) and dried at room temperature. The adherent bacterial cells were fixed with 200 µL of methanol for 15 min, then the plates were emptied and dried overnight. The adherent cells were stained with 1% crystal violet for 10 min and were washed twice with water. The dye bound to the adherent cells was dissolved with 150 mL of 95% ethanol and optical density (OD) was measured at 590 nm using a spectrophotometer (SpectroStar Nano BMG Labtech). The uninoculated wells containing BHI served as negative control. Blank corrected absorbance values of isolates were used for reporting biofilm production. Isolates were considered biofilm producers when their OD values were three times greater than the standard deviation of the mean Dc. Additionally, isolates showing an ability to produce biofilm were classified as weak (Dc < OD ≤ 2* Dc), moderate (2*Dc < OD ≤ 4* Dc) or strong (OD > 4* Dc) biofilm.

### 2.5. Whole-Genome Sequencing and Assembly

Genomic DNA was extracted from shrimp-related *S. aureus* isolates using a genomic DNA extraction kit (Magen Biotech, Guangzhou, China) according to the manufacturer’s instructions. Each DNA sample was fragmented into 400 bp fragments by a Covaris M200 sonicator and prepared for sequencing with the Ion Plus Fragment Library Kit (Thermo Fisher Scientific Inc, Waltham, MA, USA). Whole genomes were sequenced on the Life Ion S5 platform with an average coverage of 100×. Clean reads were used for de novo assembly with SPAdes v3.6.2. Only assemblies that harbored ≥ 95% of cg MLST targets were used for further analysis as previously described. If the criteria were not met, the sample was re-sequenced.

### 2.6. Determination of STs, Spa Types, SCCmec Types, Virulence Genes and ARG Genes

The STs of *S. aureus* was based on 7 housekeeping genes (*arcC*, *aroE*, *glpF*, *gmk*, *pta*, *tpi* and *yqil*) using multilocus sequence typing [23]. In order to obtain allelic profiles and to determine STs, the full-length sequences of these 7 genes from shrimp-related *S. aureus* genomes were obtained using BLAST + 2.5.0 [24] and compared at each locus with those of the known alleles in the *S. aureus* MLST database (https://pubmlst.org/saureus, accessed on 28 March 2023). *Spa* types (t) were predicted using spaTyper v1.0 webserver [25] from the Centre of Genomic Epidemiology (https://cge.cbs.dtu.dk/services/spatyper, accessed on 25 October 2022). SCC*mec* types for MRSA isolates were predicted using SCCmecFinder v1.2 from the Center of Genomic Epidemiology (https://cge.cbs.dtu.dk/services/SCCmecFinder/, accessed on 17 November 2022). The presence of virulence factors and antibiotic resistance factors encoded in the genomes were inferred by comparing all the proteins against the virulence factor database (VFDB) [26], the comprehensive antibiotic resistance database (CARD) [27], and Resfinder [28].

### 2.7. Nucleotide Sequence Accession Numbers

The complete genomic sequences of shrimp-related *S. aureus* isolates were deposited in the Foodborne *Staphylococcus aureus* genome database by the Institute of Microbiology, Guangdong Academy of Sciences (http://210.77.86.67/StaphylococcusAureus.html, accessed on 2 September 2022).

## 3. Results

### 3.1. Isolation and Identification of S. aureus from Shrimp in China

In total, 41 of 145 shrimp samples were coagulase-positive and 63 isolates were confirmed to be *S. aureus* using the API test. The qualitative and quantitative results of *S. aureus* positive samples are shown in Table 1. Overall, the isolation percentage of *S. aureus* from shrimp samples was 28.28% (41/145), with MPN values showing half of samples (22/41) less than 1 MPN/g, and only two samples were higher than or equal to 110 MPN/g. The prevalence of positive samples was found in 24 of the 39 Chinese cities as follows: 11% in Guangzhou, 9% in Macao, 8% in Hangzhou, Lasa and Nanjing and 6% in Beijing and Huhehaote, and the remaining positive samples were obtained from the other cities.

### 3.2. Antibiotic Susceptibility and Antibiotic-Resistant Genes (ARGs)

The resistance patterns of 63 shrimp-related *S. aureus* isolates of tested antibacterial agents are shown in Table 2. As a result, most shrimp-related *S. aureus* isolates showed moderate resistance to different concentrations of antibiotics, whereas only seven isolates were susceptible to all tested antibiotics. All isolates were sensitive to chloramphenicol, linezolid, quinupristin/dalfopristin, teicoplanin and nitrofurantoin. The phenotypic resistance profiles of the *S. aureus* isolates from shrimp were as follows: ampicillin, 85.7%; penicillin G, 85.7%; erythromycin, 47.6%; kanamycin, 22.2%; fusidic acid, 22.2%; tetracycline, 17.5%; clindamycin, 12.7%; telithromycin, 12.7%; amoxicillin/clavulanic acid, 11.1%; streptomycin, 9.5%; cefoxitin, 7.9%; ceftazidime, 7.9%; gentamicin, 6.4%; cefepime 4.8%; rifampicin, 4.8%; norfloxacin, 3.2%; ciprofloxacin, 3.2%; amikacin, 1.6%; trimethoprim/sulfamethoxazole (1:19), 1.6%. Five isolates were resistant to cefoxitin and carried *mecA* genes, which were confirmed as MRSA isolates based on an antibiotic resistance test, namely Sta705, Sta1827, Sta2404, Sta4104 and Sta4127A1. The resistance rate of 41 isolates to more than 3 types of antibiotics was 65.1% (41/63), showing multiple drug resistance, of which 5 isolates were resistant to more than 10 types of antibiotics, namely Sta705, Sta1827, Sta4104, Sta4127A1 and Sta4076A1.

**Figure 1:**
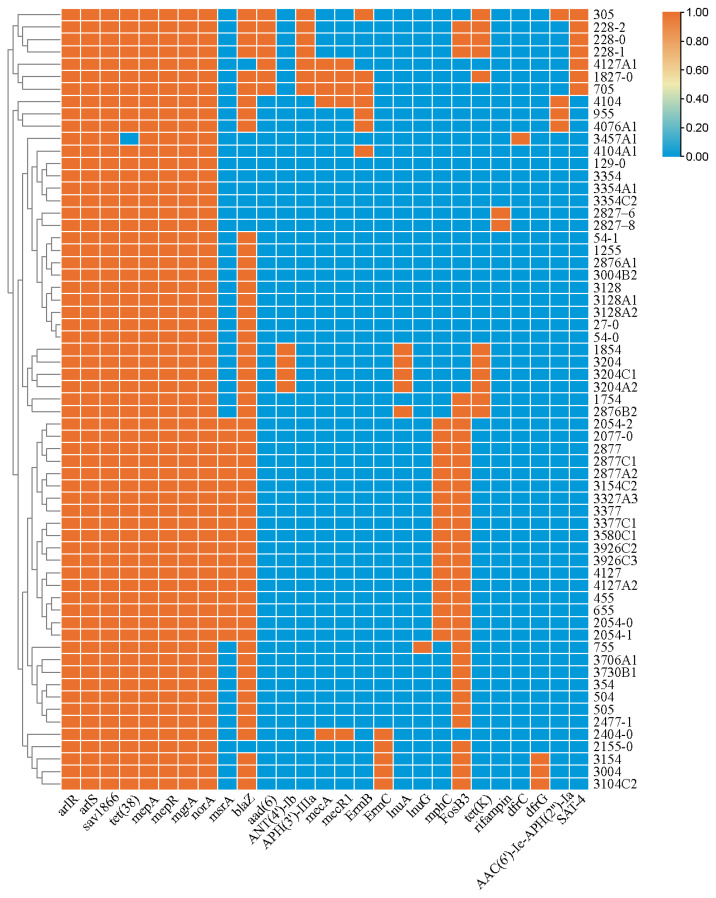
Prevalence of antibiotic-resistance-related genes in the shrimp-related *S. aureus* in China.

In this study, at least 27 known antibiotic resistance genes (ARGs) were identified (Figure 1). Each of isolates was contained 8–16 ARGs. These ARGs may confer resistance to β-lactams [*mecA* (7.9%), *mecR1* (7.9%), *blaZ* (84.1%)]; aminoglycosides [*aph(3′)-IIIa* (11.1%), *aac(6′)-le-aph(2′)-la* (6.3%), *aad(6)* (11.1%), *ANT(4′)-Ib* (6.3%); tetracycline [*tet*(K) (17.5%)]; macrolide-lincosamide-streptogramin (MLS) [*mph*(C) (28.6%), *lnu(A)* (7.9%), *lnuG* (1.6%), *ermB* (11.1%), *ermC* (7.9%)]; trimethoprim [*dfrC* (1.6%) and *dfrG* (4.8%)]; fosfomycin [*fosB* (54.0%)]; streptothricin [*SAT-4*(11.1%); and rifampin [*rpoB* (3.2%)], as well as antibiotic efflux pumps [*msr(A)* (28.6%), *mepA* (100.0%), *mepR* (100.0%), *norA* (100.0%), *mgrA* (100.0%), *tet(38)* (98.4%), *sav1866* (100.0%), *arlR* (100.0%) and *arlS* (100.0%)]. Among them, *S. aureus* 1827 carried 16 resistance genes [*arlS*, *arlR*, *mepR*, *mepA*, *tet(38)*, *blaZ, mecA*, *mecR1*, *ermB*, *aph(3′*)-*IIIa*, *SAT-4*, *aad(6)*, *tet(K)*, *mgrA*, *norA*, *sav1866*], which was resistant to 11 antibiotics in this study (AMP-FEP-FOX-PEN-CAZ-KAN-STR-CLI-ERY-TEL-TET). The comparison of resistance phenotypes and genotypes showed that the resistance phenotypes of β-lactams, tetracycline and macrolides were basically consistent with their genotypes.

### 3.3. Biofilm Production and the Presence of Biofilm-Related Genes

As showed in Table 3, all *S. aureus* strains could produce biofilm, among which 2 strains (3.2%, 2/63) had moderate biofilm formation and 61 strains (96.8%, 61/63) had strong biofilm formation as determined by MPA results. The results of *S. aureus* adhesion and biofilm-related genes are also shown in Table 3. In general, *sdrC*, *clfA* and *clfB* were found in all isolates; *sdrE*, *icaB*, *icaC*, *icaD*, *icaR*, *fnbA*, *map* and *ebp* were found in 98.4% (62/63) of isolates; *icaA* was isolated from 96.8% (61/63); *fnbB* was found in 95.2% (60/63) of isolates; *sdrD* was found in 90.5% (57/63) of isolates; *cna* gene was found in 46.0% (29/63) of isolates. Thus, the shrimp-related *S. aureus* strains harbored most of the biofilm-producing and adhesion genes. 

### 3.4. Prevalence of Virulence-Associated Genes

**Figure 2:**
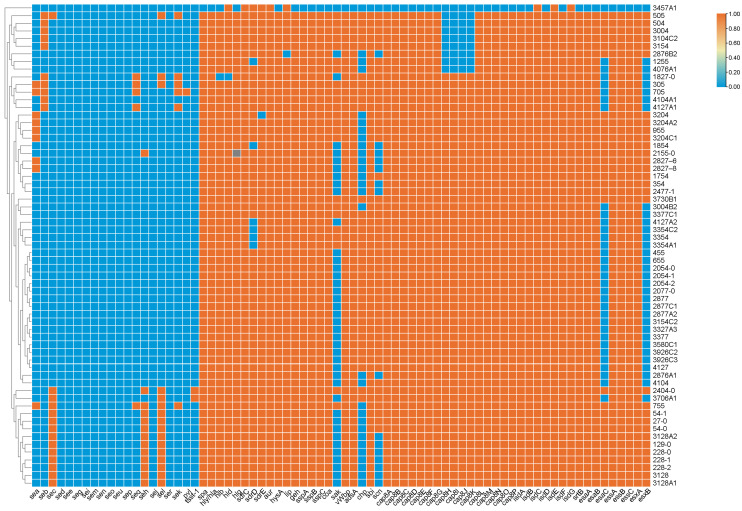
Prevalence of different virulence genes in the shrimp-related *S. aureus* in China.

As shown in Figure 2, only nine SE genes were detected in *S. aureus* isolates from shrimp, whereas *sed*, *see*, *seg*, *sei*, *sem*, *seo*, *seu*, *sep*, *sej* and *ser* were not free in the shrimp-related *S. aureus* isolates. The rates of the SEs were as follows: *sel* (25.4%), *sec* (22.2%), *seh* (20.6%), *seb* (15.9%), *sea* (14.3%), *sek* (9.5%) and *seq* (7.9%). Among the classical SE genes, 14 isolates carried *sec*, 10 isolates carried *seb*, and 9 isolates carried *sea*, whereas the *egc* gene group was not detected. Except for SE genes, the WGS analysis found 56 other VFs in *S. aureus* isolates (Figure 2). All of the conserved VFs were homologous to the VFs identified in *S. aureus*, including multiple genes related to toxin-related genes [*tsst-1* (3.2%), *pvl* (1.6%), *spa* (98%, 62/63), *hly/hla* (98%, 62/63), *hlb* (97%, 61/63), *hld* (98%, 62/63) and *hlg* (97%, 61/63)], VII secretion system regulators [*esaA* (98%,62/63), *esaB* (98%, 62/63), *esaC* (48%, 30/63), *essA* (98%, 62/63), *essB* (98%, 62/63), *essC* (98%, 62/63), *esxA* (98%, 62/63) and *esxB* (48%, 30/63)], exoenzyme [*aur* (100%, 63/63), *hysA* (98%, 62/63), *geh* (98%, 62/63), *lip* (98%, 62/63), *sspA* (98%, 62/63), *sspB* (98%, 62/63), *sspC* (98%, 62/63), *coa* (98%, 62/63), *sak* (37%, 23/63) and *vWbp* (98%, 62/63)], immunomodulatory factors [*adsA* (98%, 62/63), *chp* (54%, 34/63), *cap8A-G* (98%, 62/63), *cap8H-K* (86%, 54/63), *cap8L-P* (98%, 62/63), *sbi* (98%, 62/63) and *scn* (75%, 47/63)] and nutritional and metabolic factors [*isdA* (98%, 62/63), *isdB* (98%, 62/63), *isdC* (100%, 63/63), *isdD* (98%,62/63), *isdE* (100%, 63/63), *isdF* (98%, 62/63), *isdG* (100%, 63/63) and *srtB* (98%, 62/63)].

### 3.5. Molecular Typing of Shrimp-Related S. aureus Isolates

The genetic diversity of MLST in 63 isolates from shrimp-related *S. aureus* was analyzed (Table 4). As a result, ST15 was detected in 18 strains (28.6%), followed by ST1 (12/63, 19.0%), ST188 (7/63, 11.1%), ST7 (5.63, 7.9%), ST6 (4/63, 6.3%), ST25 (4/63, 6.3%), ST59 (4/63, 6.3%) and ST398 (3/63, 4.8%). ST4071 was detected in two isolates, and the other isolates were ST72, ST630, ST2205 and ST4692, which were single strains. Among them, ST4692 is a new ST type. The results of *Spa* typing were like those of MLST. The study found 21 different *spa* types, with the most common being t085 (27.0%), followed by t127 (14.3%), t189 (11.1%), t437 (6.3%), t701 (6.3%), t091 (4.8%), t034 (3.2%), t17886 (3.2%), t377 (3.2%) and t5837 (3.2%). Individual types were t078, t114, t1689, t17887, t17888, t258, t3033, t3092, t4309, t571 and T796. Among them, t17886, t17887 and t17888 were new *spa* types. In this study, ST15-t085 (17/63), ST1-t127 (9/63) and ST188-t189 (7/63) were the dominant genetic types. ST1-t127 was mainly found in the cities of Guangzhou (6/9) and Huhehaote (3/9). All the *S. aureus* isolates from Beijing belonged to ST15-t085.

## 4. Discussion

As is known, microbial hazards have become one of the most important threats in the field of food safety. Shrimp products are an important food source for many coastal countries in the world. *S. aureus* and its toxins can cause food poisoning and pose a risk to public health, whether it is due to source pollution in shrimp products, secondary contamination during processing and transportation or cross-contamination in food service links. In our previous study, it was found that 35.0% of retail meat samples were positive for *S. aureus* in China [29]. In this study, the contamination rate of *S. aureus* in shrimp products was 28.3%, which showed that the shrimp product was also an important resource for foodborne *S. aureus*. Fortunately, most of the positive samples had less than 1 MPN/g.

Overall, our result was higher than the results of several years ago in China. In 2013, the prevalence of *S. aureus* in Shanghai city was 4%; in 2015, the prevalence of *S. aureus* in Liaoning Province was 5.56%; and in 2016, the prevalence of *S. aureus* in Huai’an city was 18.2%. Compared with studies from other countries, our result was not lower as well. In Iran, the contamination rate of *S. aureus* in shrimp was 24.6% [30]. In Nigeria, the percentage of *S. aureus*-positive samples in ready-to-eat shrimp accounted for 30.97% [31]. The detection rate of *S. aureus* in prawns which were taken from supermarkets in and around Cochin was high (26.7%) [32]. However, all these reports were subject to district restriction. Therefore, this survey was more full-scale and systematical investigation for the prevalence of *S. aureus* isolated from shrimp products, especially as the collection site covered most provincial capitals of China and the samples were more country-specific. However, the existing data and our study indicate that *S. aureus* isolated from shrimp products in China is potentially harmful and deserves our attention. In view of the characteristics of shrimp and other aquatic products, many people like to eat shrimp raw or pickled to taste the freshness of the food itself; once contaminated by *S. aureus*, the generation of enterotoxin often easily leads to the occurrence of food poisoning events.

Staphylococcal enterotoxins (SEs) are responsible for most staphylococcal food poisoning outbreaks [33]. They can still maintain their biological and immune activity after being treated at 100 °C for 30 min. Currently, there are at least 28 kinds of SEs reported, which are mainly divided into classical enterotoxins (*sea-see*) and novel enterotoxins [34], of which classical enterotoxins (*sea-see*) account for more than 95% of confirmed food poisoning cases as the most common enterotoxin. This study examined 23 SE genes in *S. aureus* isolates that were associated with shrimp products. The results showed that 47.6% of the isolates carried one or more classical SE genes and non-classic SE genes. This suggests a potential risk of *S. aureus* in shrimp products in China. This rate was like previous research which tested for the prevalence of 18 SE genes of *S. aureus* isolates from different origins in China, showing that 54.4% of isolates harbored SE genes. In China, *sea* was the most common gene which was responsible for SFP outbreaks, followed by *seb* and *sed,* which were responsible for most outbreaks [35]. *Sed*, *sea* and *seb* were detected in 14.3% and 15.9% of *S. aureus* isolates in this study, which indicated that these shrimp-related *S. aureus* isolates have a potential risk of foodborne infections. Fortunately, the highest detection rate of classical SE genes in shrimp-related isolates was in *sec* (22.22%). However, there were also some outbreaks which have involved *sec* [36]. The common detection method of SEC was determined using immuno-colloidal gold chromatographic test strips, but the anti-SEC antibody was produced based on SEC1 or SEC2. Thus, this may have caused inaccuracy in SEC detection in previous studies. Additionally, the SFP outbreaks in recent years should also remind people not to ignore the harm caused by new enterotoxins [34]. In this study, *seq, seh, sej, sel* and *sek* were detected in *S. aureus* isolates. Of these enterotoxins, only SEH-producing strains have clearly been involved in SFP outbreaks [37,38,39], but results from different researchers have shown the high incidence of genes encoding new SEs and SE*l*s among food-borne *S. aureus* [33,40]. It is possible that the corresponding SEs might have been the cause of these outbreaks. Thus, the hazard of these isolates which harbored SE genes should not be ignored.

Except SE genes, VFs include diverse functions (e.g., adhesion, invasion, signaling, conjugation, environmental interaction, immune evasion, etc.) which play a crucial role in cell viability, virulence and evasion of host defenses [41]. In this study, two *S. aureus* isolates (Sta2404-0 and Sta3706A1) were harbored in the *tsst-1* gene. Importantly, previous studies have shown that TSST-1 is a super-antigen that can cause multiple organ dysfunction syndrome and is responsible for an especially high rate of mortality [42,43]. Moreover, the *tsst-1* gene, together with *sec* and *sel*, have often been found in *Staphylococcus aureus* Pathogenicity Islands (SaPIs) and play an important role in toxin production [33,42]. Except for TSST-1, one MRSA isolate, Sta705, carried the Panton–Valentine leukocidin (PVL) gene. In recent years, PVL-positive *S. aureus* has received a significant amount of attention [44]. These strains can cause highly necrotizing pneumonia, necrotizing dermatitis, and other primary diseases in humans [44,45]. In fact, the risk of death associated with PVL-positive *S. aureus* has been reported to be higher than that associated with non-PVL-producing *S. aureus* [46]. However, either *tsst-1* or PVL were associated with the *S. aureus* mobile genetic element (SaPIs or bacteriophages, etc.), which may promote the virulence of *S. aureus* and participate in the pathological process of *S. aureus* infection in vitro and in vivo [42,47]. The presence of TSST-1-positive *S. aureus* isolates and PVL-positive *S. aureus* isolates in shrimp samples is concerning, as they both contain classical SE genes. This poses a potential risk of foodborne infections for consumers and requires further investigation. Therefore, it is important for the public to be aware of these findings and for further attention to be given to *S. aureus* in shrimp.

As is known, the presence of biofilm on food contact surfaces is considered a health hazard. In fact, *S. aureus* biofilm on food contact surfaces poses a serious risk of food contamination [48]. It has been frequently found on surfaces of food processing plants responsible for outbreaks related to the consumption of fresh and processed foods worldwide [49,50,51,52]. Overall, the rate of biofilm producer isolates in the present study was higher than previous reports of all *S. aureus* isolates obtained from shrimp samples which showed the ability to produce biofilm with moderate or strong biofilm production capability [20,53,54,55,56]. In addition, through whole-genome analysis, it was found that all these isolates carry biofilm-related genes, including *ica* genes (*icaA, icaB, icaC, icaD* and *icaR*) and another set of adhesion genes, such as *fnbA*, *fnbB*, *cna*, *map* and *ebp*, as well as *clfA* and *clfB*. These genes are both important factors contributing to the initiation of foreign body infection [57,58,59]. Although the frequencies of detection of genes associated with adhesion and biofilm formation reported in *S. aureus* isolates from food vary according to the geographical area studied, *S. aureus* isolates from humans found that 100% of them presented these genes and were able to form biofilm [60,61]. Thus, it is necessary to continuously observe the biofilm-forming ability and related genes of *S. aureus* isolates in food.

In addition, the antimicrobial resistance was tested in shrimp-associated *S. aureus* in this study. However, many reports have detected antibiotic-resistant strains of *S. aureus* in various food products [62,63,64], and our data were also not lower. In this study, the rate of multiple drug resistance even reached 65.1%, of which five isolates resisted more than 10 antibiotics. This result was higher than many previous studies: the multidrug resistance rate of *S. aureus* isolates from aquatic products was 47.9%, from dairy cows was 38.46% and from fresh pork was 50.7%. Interestingly, five isolates resisted more than 10 antibiotics, of which four isolates were confirmed as MRSA. The MRSA isolates showed a broader range of antimicrobial resistance than MSSA. This indicates that resistance to methicillin is just the first step of a multidrug resistance process fostered by the great ability of MRSA to evade antibiotic therapy [65].

Among the different subtyping methods available, both MLST and *spa* typing have shown a significantly clonal population structure for *S. aureus*. In this study, most genotypes of *S. aureus* were related to SPF and clinic infection, such as ST1-t127, ST7-t091, ST6-t701 and ST188-t189 [66,67,68,69], which indicates that these strains of these STs have at least a theoretical pathogenic potential. ST15 was the predominant MLST type of isolate in our study. This lineage is not often listed among the major STs found in humans. In previous studies, it was identified in 2/15 healthy ST15 *S. aureus* gut carriers in a Spanish population [70], and it was one of the commonest STs among nasal *S. aureus* strains from children in Ghana [71]. In addition, ST188 and ST6 were identified in 11.1% and 6.3% of isolates in this study, respectively. In China, *S. aureus* ST6 is the predominant lineage isolated from outbreaks in Shenzhen, Xi’an and Ma’anshan, whereas ST188 has been increasingly linked to hospital-associated infections and community-associated infections, which may be one of the clones that are associated with SFP [68,72,73]. Fortunately, ST15 (28.6%), ST188 (11.1%) or ST6 (6.3%) were free of any SEs (Table 4). Interestingly, all ST1 isolates harbored the *sec* gene, whereas most ST7 and ST25 isolates harbored the *sea* or *seb* genes (Table 4). This showed that different genetic lineages might have different virulence characteristics. In China, Lv et al. (2021) have found that ST59 was the most prevalent type (32.1%, *n* = 18) in SFP isolates [69]. Interestingly, this type of isolate was the predominant CA-MRSA lineage in Asia [74,75,76]. In our study, we found that ST59-t437 was the most isolated type of MRSA. Furthermore, all the ST59-t437 MRSA isolates displayed a wider range of antimicrobial resistance and contained classical SE genes *sea* or *seb*. This suggests that these isolates have a greater likelihood of causing food poisoning.

In summary, our results provide a comprehensive information on the genetic background of shrimp-derived *S. aureus* in Chinese aquatic samples. In our study, the contamination level of *S. aureus* isolated from shrimp was significantly higher than that of other studies, indicating an increased potential contamination risk of *S. aureus* in shrimp products. These isolates were potentially virulent, and nearly half of them carried virulence factors such as enterotoxin genes. The study of drug resistance could help identify the best treatment after a food poisoning incident. All the isolates had moderate or strong biofilm-producing capacity, and most of the isolates had biofilm-related genes, indicating that these potential virulence genes could persist in food production and circulation chains. In particular, ST15, ST188 and ST6 were found for the first time to be a retained type in shrimp in this study, which should be drawn to public attention.

## Figures and Tables

**Table 1:** Distribution of *S. aureus* in retail shrimp in China.

Positive Number	Positive Sample	Sampling Site	Quantitative Methods	Qualitative Methods
			MPN Values (MPN/g)	
1	YXJ27	Guangzhou	2.3	+
2	ZCC54	Guangzhou	24	+
3	CHJ129	Guangzhou	0.36	+
4	PYJ228	Guangzhou	>110	+
5	SGC305	Shaoguan	0.74	+
6	ZJC354	Zhanjiang	110	+
7	HYC455	Heyuan	2.1	+
8	ZJC1754	Zhanjiang	3.6	+
9	SGJ1827	Shaoguan	1.1	+
10	HYC1854	Heyuan	1.5	+
11	HKC504	Haikou	0.36	+
12	HKC505	Haikou	0.92	+
13	NNC655	Nanning	4.3	+
14	FZC705	Fuzhou	0.36	+
15	XMC755	Xiamen	0.36	+
16	HKC2404	Haikou	0.36	+
17	SYJ2477	Sanya	0.62	+
18	WHC955	Wuhan	0.36	+
19	TYC1255	Taiyuan	0.36	+
20	LZC2155	Lanzhou	0.36	+
21	BJC2054	Beijing	2	+
22	BJJ2077	Beijing	2.3	+
23	CSJ2827	Changsha	<0.3	+
24	HZJ2876	Hangzhou	0.74	+
25	HZJ2877	Hangzhou	<0.3	+
26	XNC3004	Xining	0.3	+
27	HHHTC3104	Huhehaote	0.3	+
28	HHHTJ3128	Huhehaote	24	+
29	SYC3154	Shenyang	0.3	+
30	NJC3204	Nanjing	24	+
31	ZZC3327	Zhengzhou	0.92	+
32	LSC3354	Lasa	4.3	+
33	LSJ3377	Lasa	2.3	+
34	AMC3457	Macau	0.36	+
35	CSJ3580	Changsha	2.3	+
36	NJS3706	Nanjing	2.3	+
37	NJJ3730	Nanjing	0.36	+
38	ZZJ3926	Zhengzhou	1.1	+
39	CCJ4076	Changchun	0.36	+
40	AMC4104	Macau	0.36	+
41	AMJ4127	Macau	24	+

**Table 2:** Antimicrobial susceptibility tests for *S. aureus* isolates obtained from shrimp in China.

	Antibiotics	Zone Diameters (mm)			*S. aureus* (*n* = 64)		
		R	I	S	NO. of Resistant Strains (%)	NO. of Intermediate-Resistance Strains (%)	NO. of Susceptible (%)
β-Lactams	Amoxycillin/clavulanic acid	≤19	-	≥20	7 (11.1%)	0 (0%)	56 (88.9%)
	Ampicillin	≤28	-	≥29	54 (85.7%)	0 (0%)	9 (14.3%)
	Cefepime	≤14	15–17	≥18	3 (4.8%)	2 (3.2%)	58 (92.0%)
	Cefoxitin	≤21	-	≥22	5 (7.9%)	0 (0%)	58 (92.1%)
	Penicllin G	≤28	-	≥29	54 (85.7%)	0 (0%)	9 (14.3%)
	Ceftazidime	≤14	15–17	≥18	5 (7.9%)	4 (6.4%)	54 (85.7%)
Aminoglycosides	Amikacin	≤14	15–16	≥17	1 (1.6%)	14 (22.2%)	48 (76.2%)
	Gentamicin	≤12	13–14	≥15	4 (6.4%)	0 (0%)	59 (93.6%)
	Kanamycin	≤13	14–17	≥18	14 (22.2%)	10 (15.9%)	39 (61.9%)
	Streptomycin	≤11	12–14	≥15	6 (9.5%)	45 (71.4%)	12 (19.1%)
Phenicols	Chloramphenicol	≤17	18–20	≥21	0 (0%)	17 (27.0%)	46 (73.0%)
Lincosamides	Clindamycin	≤14	15–20	≥21	8 (12.7%)	7 (11.1%)	48 (76.2%)
Macrolides	Erythromycin	≤13	14–22	≥23	30 (47.6%)	5 (7.9%)	28 (44.5%)
	Telithromycin	≤18	19–21	≥22	8 (12.7%)	10 (15.9%)	45 (71.4%)
Fluoroquinolones	Ciprofloxacin	≤15	16–20	≥21	2 (3.2%)	3 (4.8%)	58 (92.0%)
	Norfloxacin	≤12	13–16	≥17	2 (3.2%)	3 (4.8%)	58 (92.0%)
Tetracyclines	Tetracycline	≤14	15–18	≥19	11 (17.5%)	0 (0%)	52 (82.5%)
Oxazolidinones	Linezolid	≤20	-	≥21	0 (0%)	0 (0%)	63 (100%)
Ansamycins	Rifampicin	≤16	17–19	≥20	3 (4.8%)	1 (1.6%)	59 (93.6%)
Sulfonamides	Trimethoprim/sulphamethoxazole 1:19	≤10	11–15	≥16	1 (1.6%)	1 (1.6%)	61 (96.8%)
Quinolones	Quinupristin/dalfopristin	≤15	16–18	≥19	0 (0%)	3 (4.8%)	60 (95.2%)
Glycopeptides	Teicoplanin	≤10	11–13	≥14	0 (0%)	16 (25.4%)	47 (74.6%)
Nitrofurantoins	Nitrofurantoin	≤14	15–16	≥17	0 (0%)	6 (9.5%)	57 (90.5%)
	Fusidic acid	≤24	-	≥25	14 (22.2%)	0 (0%)	49 (77.8%)

**Table 3:** Biofilm formation ability and biofilm-associated genes in *S. aureus* strain isolates from shrimp.

No.	*S. aureus* Isolates	Biofilm Production Assay *	Biofilm Production Ability *	Biofilm-Producing Genes					Adhesion Genes									
				*icaA*	*icaB*	*icaC*	*icaD*	*icaR*	*clfA*	*clfB*	*fnbA*	*fnbB*	*cna*	*map*	*ebp*	*sdrC*	*sdrD*	*sdrE*
1	27-0	3.5427	+++	+	+	+	+	+	+	+	+	+	+	+	+	+	+	+
2	54-0	2.6298	+++	+	+	+	+	+	+	+	+	+	+	+	+	+	+	+
3	54-1	2.9668	+++	+	+	+	+	+	+	+	+	+	+	+	+	+	+	+
4	129-0	2.9715	+++	+	+	+	+	+	+	+	+	+	+	+	+	+	+	+
5	228-0	2.8057	+++	+	+	+	+	+	+	+	+	+	+	+	+	+	+	+
6	228-1	3.3775	+++	+	+	+	+	+	+	+	+	+	+	+	+	+	+	+
7	228-2	2.1027	+++	+	+	+	+	+	+	+	+	+	+	+	+	+	+	+
8	305	1.2128	+++	+	+	+	+	+	+	+	+	+	-	+	+	+	+	+
9	354	1.8072	+++	+	+	+	+	+	+	+	+	+	+	+	+	+	+	+
10	455	2.9610	+++	+	+	+	+	+	+	+	+	+	-	+	+	+	+	+
11	1754	1.1967	+++	+	+	+	+	+	+	+	+	+	+	+	+	+	+	+
12	1827-0	2.0557	+++	+	+	+	+	+	+	+	+	+	-	+	+	+	+	+
13	1854	2.9648	+++	+	+	+	+	+	+	+	+	+	-	+	+	+	-	+
14	504	2.5967	+++	+	+	+	+	+	+	+	+	+	-	+	+	+	+	+
15	505	1.0083	++	+	+	+	+	+	+	+	+	+	-	+	+	+	+	+
16	655	2.4525	+++	+	+	+	+	+	+	+	+	+	-	+	+	+	+	+
17	705	2.8222	+++	+	+	+	+	+	+	+	+	-	-	+	+	+	+	+
18	755	3.0410	+++	+	+	+	+	+	+	+	+	+	+	+	+	+	+	+
19	2404-0	2.2565	+++	+	+	+	+	+	+	+	+	+	+	+	+	+	+	+
20	2477-1	1.7952	+++	+	+	+	+	+	+	+	+	+	+	+	+	+	+	+
21	955	2.1990	+++	+	+	+	+	+	+	+	+	+	-	+	+	+	+	+
22	1255	2.7460	+++	+	+	+	+	+	+	+	+	+	+	+	+	+	-	+
23	2155-0	3.4222	+++	+	+	+	+	+	+	+	+	+	+	+	+	+	+	+
24	2054-0	1.3443	+++	+	+	+	+	+	+	+	+	+	-	+	+	+	+	+
25	2054-1	1.8343	+++	+	+	+	+	+	+	+	+	+	-	+	+	+	+	+
26	2054-2	1.3802	+++	+	+	+	+	+	+	+	+	+	-	+	+	+	+	+
27	2077-0	2.2180	+++	+	+	+	+	+	+	+	+	+	-	+	+	+	+	+
28	2827–6	1.0738	++	+	+	+	+	+	+	+	+	+	+	+	+	+	+	+
29	2827–8	1.4532	+++	+	+	+	+	+	+	+	+	+	+	+	+	+	+	+
30	2876A1	1.5003	+++	+	+	+	+	+	+	+	+	+	+	+	+	+	+	+
31	2876B2	2.0088	+++	-	-	-	-	-	+	+	+	+	-	+	+	+	+	+
32	2877	3.4300	+++	+	+	+	+	+	+	+	+	+	-	+	+	+	+	+
33	2877C1	3.4535	+++	+	+	+	+	+	+	+	+	+	-	+	+	+	+	+
34	2877A2	2.1013	+++	+	+	+	+	+	+	+	+	+	-	+	+	+	+	+
35	3004	2.3895	+++	+	+	+	+	+	+	+	+	+	-	+	+	+	+	+
36	3004B2	2.1682	+++	+	+	+	+	+	+	+	+	+	+	+	+	+	+	+
37	3104C2	2.5065	+++	+	+	+	+	+	+	+	+	+	-	+	+	+	+	+
38	3128	2.2482	+++	+	+	+	+	+	+	+	+	+	+	+	+	+	+	+
39	3128A1	2.8780	+++	+	+	+	+	+	+	+	+	+	+	+	+	+	+	+
40	3128A2	2.4757	+++	+	+	+	+	+	+	+	+	+	+	+	+	+	+	+
41	3154	2.3555	+++	+	+	+	+	+	+	+	+	+	-	+	+	+	+	+
42	3154C2	3.5033	+++	+	+	+	+	+	+	+	+	+	-	+	+	+	+	+
43	3204	3.2423	+++	+	+	+	+	+	+	+	+	+	-	+	+	+	+	-
44	3204C1	3.3025	+++	+	+	+	+	+	+	+	+	+	-	+	+	+	+	+
45	3204A2	3.1310	+++	+	+	+	+	+	+	+	+	+	-	+	+	+	+	+
46	3327A3	3.5470	+++	+	+	+	+	+	+	+	+	+	-	+	+	+	+	+
47	3354	2.5655	+++	+	+	+	+	+	+	+	+	+	+	+	+	+	-	+
48	3354A1	2.8357	+++	+	+	+	+	+	+	+	+	+	+	+	+	+	-	+
49	3354C2	2.2680	+++	+	+	+	+	+	+	+	+	+	+	+	+	+	-	+
50	3377	2.4502	+++	+	+	+	+	+	+	+	+	+	-	+	+	+	+	+
51	3377C1	2.0077	+++	+	+	+	+	+	+	+	+	+	+	+	+	+	+	+
52	3457A1	3.7317	+++	+	+	+	+	+	+	+	-	-	-	-	-	+	+	+
53	3580C1	3.2920	+++	+	+	+	+	+	+	+	+	+	-	+	+	+	+	+
54	3706A1	3.6107	+++	-	+	+	+	+	+	+	+	+	-	+	+	+	+	+
55	3730B1	1.3638	+++	+	+	+	+	+	+	+	+	+	+	+	+	+	+	+
56	3926C2	2.1268	+++	+	+	+	+	+	+	+	+	+	-	+	+	+	+	+
57	3926C3	2.6023	+++	+	+	+	+	+	+	+	+	+	-	+	+	+	+	+
58	4076A1	1.7465	+++	+	+	+	+	+	+	+	+	-	+	+	+	+	+	+
59	4104	2.3777	+++	+	+	+	+	+	+	+	+	+	+	+	+	+	+	+
60	4104A1	2.6428	+++	+	+	+	+	+	+	+	+	+	+	+	+	+	+	+
61	4127	2.9718	+++	+	+	+	+	+	+	+	+	+	-	+	+	+	+	+
62	4127A1	3.1565	+++	+	+	+	+	+	+	+	+	+	-	+	+	+	+	+
63	4127A2	2.3085	+++	+	+	+	+	+	+	+	+	+	-	+	+	+	-	+

* Quantification of biofilm formation by optical density (OD) analysis: (+++): strong biofilm producers (OD590 > 1.68), (++): moderate biofilm producers (1.68 > OD590 > 0.84), (+): weak biofilm producers (0.84 > OD590 > 0.42). In the gene table, symbol “-” indicates the gene is not available in the strain, symbol “+” indicates the gene is available in the strain.

**Table 4:** The genetic diversity of MLST and *spa*-type of *S. aureus* isolates from shrimp.

No.	Strains	ST	*spa*-Type	Sample Origin	Antibiotic Profiles	SEs Gene
	27-0	1	t5837	Guangzhou	AMP-PEN	*sec*-*seh*-*sel*
2	54-0	1	t127	Guangzhou	AMP-PEN	*sec*-*seh*-*sel*
3	54-1	1	t127	Guangzhou	AMP-PEN	*sec*-*seh*-*sel*
4	129-0	1	t127	Guangzhou	-	*sec*-*seh*-*sel*
5	228-0	1	t127	Guangzhou	AMP-PEN--KAN-TET-RIF	*sec*-*seh*-*sel*
6	228-1	1	t127	Guangzhou	AMP-PEN--KAN-TET-RIF	*sec*-*seh*-*sel*
7	228-2	1	t127	Guangzhou	AMP-PEN--KAN-TET-RIF	*sec*-*seh*-*sel*
8	305	59	t437	Shaoguan	AMP-PEN--KAN-STR-CLI-ERY-TEL-TET-FD	*sea*-*seb*-*sek*-*seq*
9	354	6	t701	Zhanjiang	AMP-PEN	-
10	455	15	t4309	Heyuan	AMP-PEN-ERY	-
11	1754	6	t701	Zhanjiang	AMP-PEN	-
12	1827-0	59	t437	Shaoguan	AMP-FEP-FOX-PEN-CAZ-KAN-STR-CLI-ERY-TEL-TET	*seb*-*sek*-*seq*
13	1854	7	t091	Heyuan	AMP-PEN-KAN-TET	-
14	504	25	t17887	Haikou	AMP-PEN	*seb*
15	505	25	t078	Haikou	AMP-PEN	*seb*-*sec*-*sel*-*sek*
16	655	15	t085	Nanning	AMP-PEN-ERY	-
17	705	59	t437	Fuzhou	AMC-AMP-FOX-PEN-CAZ-KAN-STR-CLI-ERY-TEL	*sea*-*seb*-*sek*-*seq*
18	755	1	t5837	Xiamen	AMP-PEN	*sea*-*sec*-*seh*-*sel*-*sek*-*seq*
19	2404-0	1	t114	Haikou	AMC-AMP-FEP-FOX-PEN-CAZ-ERY	*sec*-*seh-sel*
20	2477-1	6	t701	Sanya	AMP-PEN	-
21	955	7	t796	Wuhan	AMP-PEN-GEN-KAN-CLI-ERY-TEL-FD	*sea*
22	1255	398	t571	Taiyuan	AMP-PEN-FD	-
23	2155-0	398	t034	Lanzhou	ERY	*seh*
24	2054-0	15	t085	Beijing	AMP-PEN-ERY	-
25	2054-1	15	t085	Beijing	AMP-PEN-ERY	-
26	2054-2	15	t085	Beijing	AMP-PEN-ERY	-
27	2077-0	15	t085	Beijing	AMP-PEN-ERY	-
28	2827–6	4071	t17886	Changsha	——	*sea*
29	2827–8	4071	t17886	Changsha	——	*sea*
30	2876A1	188	t189	Hangzhou	AMC-AMP-PEN-FD	-
31	2876B2	630	t377	Hangzhou	AMP-PEN-TET-FD	-
32	2877	15	t085	Hangzhou	AMP-PEN-ERY	-
33	2877C1	15	t085	Hangzhou	AMP-PEN-ERY	-
34	2877A2	15	t085	Hangzhou	AMP-PEN-ERY	-
35	3004	2205	t377	Xining	AMC-AMP-PEN-ERY-CIP-NOR-TET	*seb*
36	3004B2	188	t189	Xining	AMP-PEN	-
37	3104C2	25	t3033	Huhehaote	AMP-PEN-ERY	*seb*
38	3128	1	t127	Huhehaote	AMP-PEN-FD	*sec-seh-sel*
39	3128A1	1	t127	Huhehaote	AMP-PEN	*sec-seh-sel*
40	3128A2	1	t127	Huhehaote	AMP-PEN	*sec-seh-sel*
41	3154	25	t258	Shenyang	AMP-PEN-ERY	*seb*
42	3154C2	15	t085	Shenyang	AMP-PEN-ERY	-
43	3204	7	t1689	Nanjing	AMP-PEN-KAN-TET	*sea*
44	3204C1	7	t091	Nanjing	AMP-PEN-KAN-TET	*sea*
45	3204A2	7	t091	Nanjing	AMP-PEN-KAN-TET	*sea*
46	3327A3	15	t085	Zhengzhou	AMP-PEN-CLI-ERY	-
47	3354	188	t189	Lasa	——	-
48	3354A1	188	t189	Lasa	——	-
49	3354C2	188	t189	Lasa	——	-
50	3377	15	t085	Lasa	AMP-PEN-ERY-FD	-
51	3377C1	15	t085	Lasa	AMP-PEN-ERY-FD	-
52	3457A1	72	t3092	Macau	——	-
53	3580C1	15	t085	Changsha	AMP-PEN-ERY	-
54	3706A1	4692	t17888	Nanjing	AMP-PEN-FD	*sec-sel*
55	3730B1	6	t701	Nanjing	AMP-PEN	-
56	3926C2	15	t085	Zhengzhou	AMP-PEN-ERY-FD	-
57	3926C3	15	t085	Zhengzhou	AMP-PEN-ERY-FD	-
58	4076A1	398	t034	Changchun	AMC-AMP-PEN-AMK-GEN-KAN-CLI-ERY-TEL-SXT	-
59	4104	188	t189	Macau	AMC-AMP-FEP-FOX-PEN-CAZ-GEN-KAN-STR-CLI-ERY-TEL-CIP-NOR	-
60	4104A1	188	t189	Macau	TEL-FD	*seb*
61	4127	15	t085	Macau	AMP-PEN-STR-ERY	-
62	4127A1	59	t437	Macau	AMC-AMP-FOX-PEN-CAZ-KAN-STR-CLI-ERY-TEL-FD	*seb-sek-seq*
63	4127A2	15	t085	Macau	AMP-PEN-ERY-FD	-

## Data Availability

The data presented in this study are available on request from the corresponding author.

## Supplementary Materials

The following supporting information can be downloaded at: https://www.mdpi.com/article/10.3390/foods12142651/s1, Figure S1: The shrimp numbers in each sampling city; Figure S2: Sampling site of Staphylococcus isolates.
