# Supplementary figures and images for: Occurrence, Antibiotic Susceptibility, Biofilm Formation and Molecular Characterization of Staphylococcus aureus Isolated from Raw Shrimp in China
Source: Foods. 2023 Jul 10;12(14):2651. doi: 10.3390/foods12142651 (PMC10378822; doi:10.3390/foods12142651)

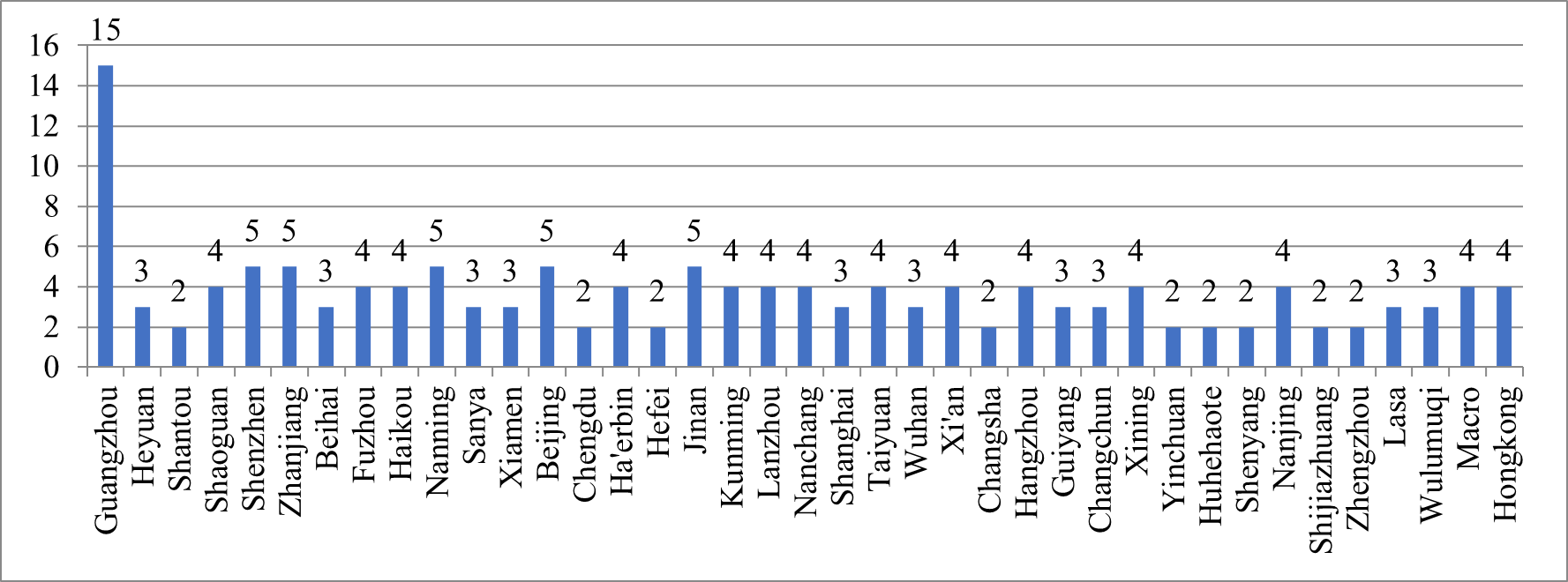

Supplement: Supplementary file 1 [file foods-12-02651-s001.zip › Figure S1 The shrimp numbers in each sampling city.png]

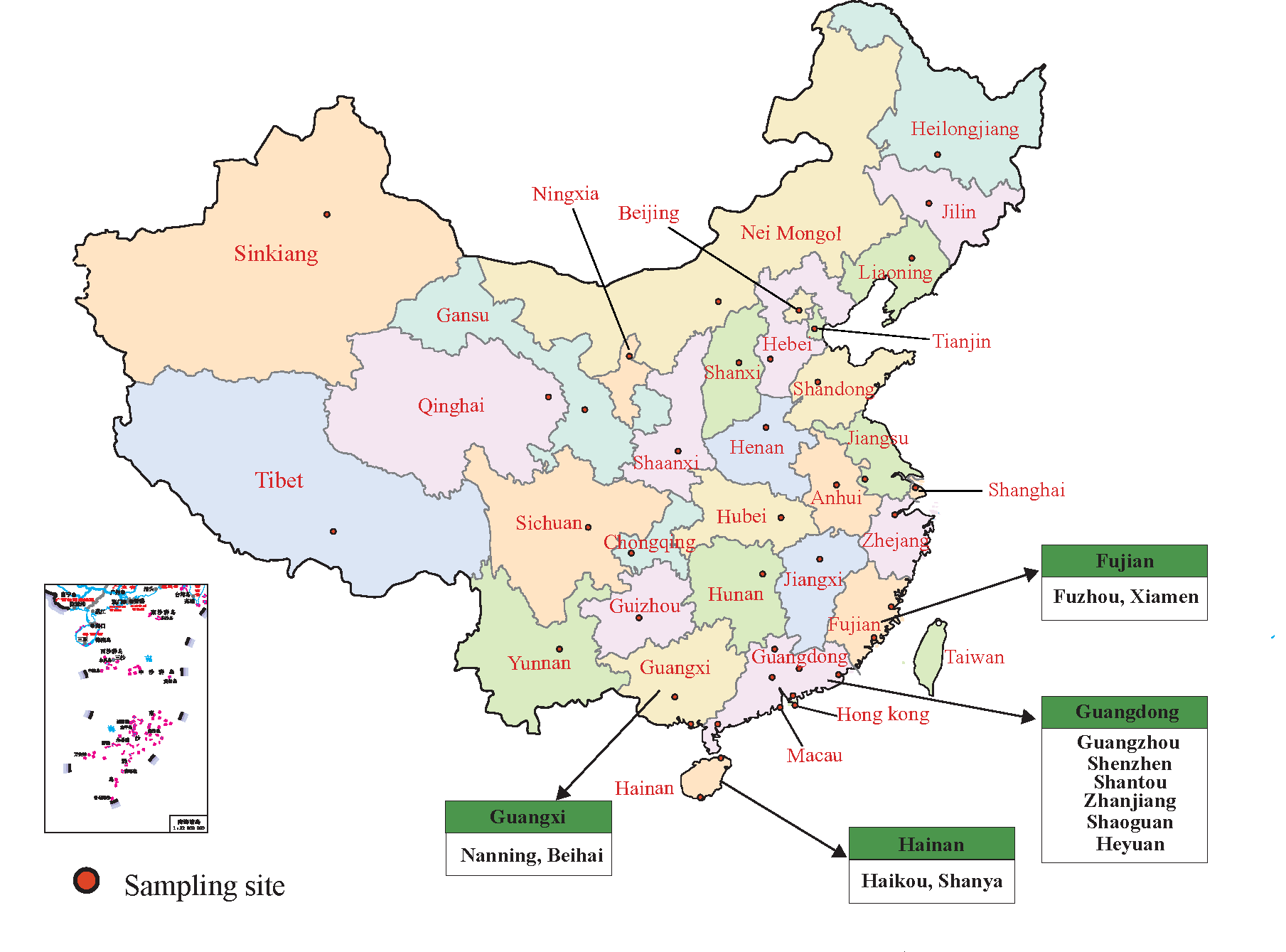

Supplement: Supplementary file 1 [file foods-12-02651-s001.zip › Figure S2 Sampling site of Staphylococcus isolates.tif]
